# City Brand Image Building and Its Impact on the Psychological Capital of New Entrepreneurs Following Cultural Construction

**DOI:** 10.3389/fpsyg.2021.717303

**Published:** 2021-11-15

**Authors:** Yingji Li, Penghang Hsü, Guanghui Hao, Kaiyang Sun, Yahong Wang

**Affiliations:** ^1^School of Management, Chongqing Institute of Engineering, Chongqing, China; ^2^Law School, East China Normal University, Shanghai, China; ^3^School of Public Management, Southwest University of Finance and Economics, Chengdu, China; ^4^Department of Management, Monash University, Melbourne, VIC, Australia; ^5^Law School, Capital University of Economics and Business, Beijing, China; ^6^Criminal Investigation Police University of China, Shenyang, China

**Keywords:** cultural construction, city brand, image design, entrepreneur, psychological capital

## Abstract

The purpose of the study is to provide effective direction and ideas for urban modernization and promote the development of the city innovation economy and the stability of the employment rate. First, the main contents and influencing factors of urban culture construction are introduced. Second, the construction of city cultural images and the social capital of new entrepreneurs are discussed, and the relationship between the two is analyzed. Then, Interpretative Structural Modeling Method (ISM) is put forward, and five influencing factors of city entrepreneurial environments are expounded. A questionnaire survey is designed based on the ISM model, and a nationwide survey of new entrepreneurs is carried out. The survey results show that entrepreneurs of different genders, ages, and educational levels have different degrees of concern for the city image. Among them, the entrepreneurs with different educational levels have the most obvious difference in their attention to the cultural image of the city (*p* < 0.05). In addition, public transportation, educational conditions, tourism resources, air quality, and image orientation are the most obvious factors affecting the construction of city brand images (*p* < 0.05). The influence of the educational level of residents and investment environment on new entrepreneurs is more prominent. This shows that in the process of shaping the city brand image, the improvement of city culture is helpful for new enterprises. The more perfect the city culture is, the more attractive it will be for highly educated entrepreneurs. The study can help relevant decision-makers to plan the future development direction of the city more accurately and realize the stable development of city brand images.

## Introduction

Culture is an important part of comprehensive national strength. As the highest level of regional cultural development, city culture is materialized into a new growth point of the national economy. A city is an advanced place for population gathering and one of the signs of human civilization (Jiang and Zhang, 2020). The city is the main carrier of culture since it came into being. The architectural style, street layout, and spatial distribution of the city have a profound impact on the history and culture of the city. With the accelerating pace of urbanization, the importance of city cultural construction is paid more and more attention by society and governments at all levels (Tao et al., 2018). The government realizes that economic, social development, and cultural development complement one another. Culture is the manifestation of modern economic society, and economic society becomes a lasting driving force to promote cultural development (Yuan and Jiang, 2018). Culture contains the connotation of the city, is the heart and lifeblood of the city, and is the foundation of the sustainable growth of the city. Therefore, improving the core competitiveness of the city by strengthening the construction of city culture is the only way for the prosperity and development of city culture in Xi’an (Lin and Jiang, 2018). With the development of urbanization, there come more and more cities. In the rapid economic development of today, the city becomes an important subject of competition. A good city image not only enhances the competitiveness of the city but also affects the future development of the city and the country (Harp, 2018). Therefore, it has practical significance to study the problem of city image. City image refers to the impression of a city in the minds of the public. It is the perception of the city by the public, including the perception and evaluation of the shape and characteristics of the city. City image includes both objective social existence and subjective evaluation.

The construction of an entrepreneurial economy not only requires the government and social departments to “refrain from doing some things to accomplish other things” at the macro policy and cultural level but also requires the rapid growth of new ventures and the continuous activation and rational allocation of entrepreneurship. If an entrepreneur stops the development and extension of cultural values in the new environment and situation, it cannot maintain sustainable innovation, that is, the enterprise cannot maintain its strong vitality, so the intrinsic quality of entrepreneurship will gradually disappear, and the cultural value will be lost. Therefore, the essence of entrepreneurship is the external expansion of the internal cultural values of entrepreneurs. In a certain social, cultural, and political atmosphere, entrepreneurial conditions affect entrepreneurial opportunities and entrepreneurial ability. Entrepreneurial opportunities and ability are synthesized in the entrepreneurial efforts to generate entrepreneurial activities or new enterprises. It is necessary to continuously improve the entrepreneurial environment conditions of cities, carry out new entrepreneurial activities, or make new enterprises bring sustainable vitality to economic growth. As the main condition affecting the entrepreneurial environment of cities, city images affect the investment behavior and development path of entrepreneurs in many ways. Wu and Wu (2019) proposed the positive psychological role in promoting enterprise innovation behavior and verified it by using the structural model (Wu and Wu, 2019). However, compared with the entrepreneurs in developed market economy countries, Chinese entrepreneurs have to play a dual role from the beginning as they are one of the main creators of the economic growth miracle of China as traditional entrepreneurs (Hashemi, 2019) and they are also an important driving force for the social transformation of China and the establishment of a market economic system as institutional entrepreneurs (Chen et al., 2019). The dual mission of specific time and space, along with the role of the main undertaker in the growth of entrepreneurial enterprises make Chinese entrepreneurs face great pressure. Qian et al. (2018) proposed the basis of a positive correlation between enterprise performance and employee feedback, reflecting the importance of the psychological capital of entrepreneurs (Qian et al., 2018). How to make Chinese entrepreneurs out of the pressure dilemma and promote the development of an entrepreneurial economy becomes a common issue faced by government departments, entrepreneurs, and scholars. On the basis of cultural construction, the main connotation of city culture, the construction content of city image, and the operation state of psychological capital of new entrepreneurs are analyzed, and the main factors influencing city brand image and the areas of attention of new entrepreneurs to city image power are explored. This has a certain reference and guiding significance for the process of urbanization and the development of innovative enterprises at this stage. The understanding and evaluation of the entrepreneurial environment are directly related to the creation of new entrepreneurial opportunities and the improvement of entrepreneurial ability, and the understanding of entrepreneurial environment conditions is the basis for the evaluation of the city entrepreneurial environment. The construction of the entrepreneurial environment evaluation model and empirical research from the perspective of theoretical research can improve the entrepreneurial theory. The systematic research on the evaluation method of entrepreneurial environment and on the impact of entrepreneurial environment on entrepreneurial opportunities and entrepreneurial ability can provide a theoretical reference for the development of entrepreneurial activities in the unique regional economic environment of China.

Based on the elements of the city entrepreneurial environment and the literature in China and foreign countries, the research model of this study is established. That is, the interpretative structural model is used to construct the adjacency matrix between the elements to calculate the reachable matrix, forming the hierarchy of Interpretative Structural Modeling Method (ISM) of the city entrepreneurial environment. According to ISM, the key elements of the city entrepreneurial environment are determined, and the single factor investigation and analysis of five major city environmental factors are carried out. Finally, the influencing factors of the city brand image and the attention of new entrepreneurs to the city image are determined. It has a reference and guiding significance for the shaping of city images and the development of new enterprises. The innovation of this manuscript is as follows: (1) the evaluation method of the entrepreneurial environment is systematically studied to explore the impact of the entrepreneurial environment on entrepreneurial opportunities and entrepreneurial capabilities; (2) the adjacency matrix between elements is constructed by using the interpretative structural model, and the hierarchical structure of the city entrepreneurial environment is formed; and (3) the single factor investigation and analysis of the environmental factors of the five cities are carried out.

## Construction of City Culture and the Development of the Psychological Capital of New Venture

### City Cultural Construction

Cultural construction is a dynamic concept, which is a process of improving and perfecting various problems in the cultural process through the construction subject according to their own development needs (Bai et al., 2020). The construction of city culture is a multidisciplinary research field, including all aspects of city culture. In theoretical circles, city cultural construction can be divided into broad and narrow senses, including city material, institutional, and spiritual construction. Based on the definition of cultural construction, city cultural construction refers to the improvement, reform, and innovation of city culture through the material level, institutional level, and spiritual level according to the needs of people. It is a higher stage of city development in the process of urban modernization after the relay production construction and public facilities construction (Yang, 2017), and a process of city branding. The theoretical research of city culture provides strong guidance for the specific practice of city cultural construction. The connotation of city cultural construction mainly includes four parts, namely, the shaping of city cultural image, the utilization of city historical and cultural resources, the development of city cultural industry, and the participation of city social members (Jin, 2017).

First, the shaping of the cultural image of the city. The literal meaning of the cultural image of the city can be understood as the cultural elements in the vision of the city. A city image is the first impression of a city. The city is carefully planned and designed in all aspects to show the image of the city, from the style of the city buildings to the building of the blocks of the city, from the placement of the city sculptures to the layout of city parks, and from the design of the city squares to the extraction of the street lights of the city. The construction of modern city image is added more cultural elements to it, which not only effectively avoids “following the same pattern,” but also makes the profound cultural heritage of some landmark buildings of the city easily understood.

Second, the utilization of city historical and cultural resources. The utilization of city historical and cultural resources includes the protection and development of historical and cultural resources. The ancient buildings, antiquities, and other historical cultural heritage of the city should be protected, and then reasonable development and utilization should be carried out. Historical and cultural resources are valuable assets for a city, and their reasonable utilization can more clearly interpret the overall style of a city (Shan, 2017).

Third, the development of the cultural industry of the city. The development of the cultural industry of the city should be determined according to the specific situation of the current development of each city. Choosing the cultural industry development suitable for their city is conducive to increasing the selectivity of city cultural construction. For example, Tokyo, Japan is in favor of the animation industry, and its animation industry is famous all over the world. As an emerging industry, the rapid development of the city cultural industry opens up the gap between cities. Therefore, the development of cultural industry should grasp the local cultural resources with distinctive characteristics and learn from the excellent experience in China and foreign countries in the process of transformation and utilization, achieving better development.

Fourth, the participation of the members of society. The participation of the members of society is a key step in the construction of city culture. The strong participation of the members of society is conducive to the collection of opinions from various parties, and the results are the common results of various forces. In the process of city cultural construction, the participation of the members of society should be fully considered. In the process of participation of the members of society, the overall quality of citizens should be fully cultivated. As one of the main bodies of city cultural construction, the quality of citizens also has a corresponding impact on city cultural construction (Xie et al., 2020). In terms of city development, the economy precedes culture. The main features of modern city cultural construction are as follows. First, highlighting historical and cultural features (Datu et al., 2018). The ancient style and charm of a city is the mark of development for thousands of years, carrying and witnessing history. Second, showing the style of contemporary culture. Different cultures are combined to show the contemporary city cultural construction in an all-around way, especially for the emerging cultural industry, timely effective publicity, and interaction through online and offline media, and showing the results to everyone timely (Alessandri et al., 2018). Third, predicting the cultural trend in the future. The competition between city cultures is not only aware of the present but also the future. Therefore, in the process of construction, long-term planning should be done well, and combined with short-term planning, the unique cultural space of the city should be created. The construction of city cultural image taken as the first step, and then the historical and cultural resources of the city are excavated. The cultural industry should be selectively developed and utilized and worked together to make suggestions for the city cultural construction through the multi-party participation of the government, market, and social forces, realizing the city cultural construction (Harms et al., 2018).

Domestic scholars have three views on the concept of the entrepreneurial environment. The first is Platform theory. Some scholars believe that the connotation of entrepreneurial environment refers to a public platform built by the government and society for entrepreneurs to establish new enterprises. The entrepreneurial environment is the stage of entrepreneurial activities. The second view is the Factor theory. Entrepreneurial environment refers to the combination of various factors in the entrepreneurial process. Yang Wubin believes that the entrepreneurial environment refers to the sum of all external factors that change around the growth of entrepreneurial enterprises and can affect the growth of entrepreneurial enterprises. The city entrepreneurial environment is the aggregation of a series of concepts, which is the result of various factors. The technological entrepreneurial environment refers to the sum of direct or indirect external influencing factors related to the entrepreneurial activities of technological enterprises. The third view is concerned with both the platform and the factors. The entrepreneurial environment is the external condition of people’s entrepreneurship. It is composed of comprehensive factors. Generally speaking, domestic scholars focus on factor theories, which can provide a theoretical reference for the construction of entrepreneurial environment evaluation indexes (Lu et al., 2019; Liu et al., 2021).

Therefore, according to the above, hypothesis 1 is obtained, which is defined as H1:

H1: City social images have a positive impact on the performance of new ventures.

### Theory of City Image Building and Its Influencing Factors

The settlement state of a city reflects the social attribute of a group (Jeun and Kim, 2018), and the content of city image includes two aspects. First, the setting of all hardware systems, including the overall layout design of the city, city roads, landscaping, and environmental sanitation (Anglin et al., 2018). Second, the city software system, including government external image display, government work behavior, city civilization construction, city culture transmission, city activity establishment, quality image of city residents, city color use, and city logo design (Shiro et al., 2020). All these provide a more transmissible and infectious basis for city image perception. With the intense competition of cities, each city needs to shape its city image with distinctive city image positioning to obtain rich returns for the city (Wu and Song, 2019). It is a very important way to build a bright city image through various activities. Such activities include various natural characteristics of the city. The dimensions of city image include nature, entertainment activities, shopping, catering, commercial creativity, social capital, social relations and cohesion, cultural tolerance, cooperation and interaction, and cultural activities (Feng and Chen, 2020; Thilanhuong and Dérioz, 2020).

City cultural construction is a complex system engineering, involving planning, cooperation, and implementation. In the numerous strategies, the basic ideas are as follows. Firstly, constructing a city cultural theme. It is the premise of city cultural construction. The establishment of a city cultural theme should be refined according to the local cultural environment and the cultural resources of the city itself (Mansour, 2020). Proper construction of city cultural theme helps to clarify the development context of a city, guide the direction of city construction, shape a positive city image, enhance the popularity, taste, core competitiveness, and finally promote the sustainable development of the city (Liu and Liu, 2019). Second, clarifying the overall planning of city cultural construction. Based on the principle of “scientific planning, people-oriented,” the historical factors, realistic factors, and future factors of city culture are comprehensively explored to integrate into the historicity, reality, and future of city culture. Third, mining the cultural resources of the city. The cultural industry gradually becomes the main carrier of competition among cities. As an emerging industry, it has strong competitiveness in the current city development process. In addition, the improvement of city science and education is also conducive to the improvement of the quality of city social members, realizing the multiple co-construction of city cultural construction (Putri, 2019). Fourth, strengthening cultural publicity. It is urgent to speed up the cultural publicity of the city and shape the cultural image of the city. The publicity of city culture should fully display the image of city culture with the help of traditional media and new media (Xikui, 2020).

Therefore, hypothesis 2 is obtained and defined as H2:

H2: City cultural images have a positive impact on the performance of new ventures.

### Operation Principle of Psychological Capital in New Ventures

The so-called new venture refers to a new enterprise with innovative entrepreneurial activities as the main driving factor of economic growth (Cvp et al., 2020). Entrepreneurs and their innovation and entrepreneurship activities play a key role in the economic and social-economic form (Xia et al., 2020). The culture and policies of a region play a very important role in the development of new-venture enterprises (Yu, 2019). Wu et al. (2019) found that it was very important for entrepreneurship education and entrepreneurship practice to propose the driving factors behind the exploration and search of entrepreneurial intention and analyzed the effect of positive psychology on self-efficacy of entrepreneurship (Wu et al., 2019). Psychological capital is a kind of capital asset without physical form, which can bring economic benefits for enterprises in a long period and reflect the accumulation or accumulation results of enterprise investment activities. Human capital, social capital, tangible capital, and financial capital are the key factors to promote the successful development of enterprises. Psychological capital is the traceability of human capital and social capital, two typical intangible capital. Human capital focuses on the direction and intensity of individual involvement and emphasizes the knowledge and skills that individuals should possess. Social capital focuses on the radiation direction and coverage of the individual network, emphasizes the relationship network, and network of individuals while psychological capital is devoted to exploring the main characteristics of human capital and social capital research content. The influence of psychological capital factors permeates the process of human capital and social capital development and change mode. Positive psychological state and psychological characteristics can effectively affect the choice mode, cognitive direction, and behavior result of the individual in the process of accumulating social capital and human capital. A negative psychological state and psychological characteristics restrict the flexibility of individual thoughts and actions. The development of psychological capital can expand the conventional thinking and behavior mode of the individual, increase the node and coverage of the individual network system, and create more sustainable resources for individuals and groups. This will produce the spiral trend of individual and group growth and potential development and improve the market utility and economic benefits of social capital.

The psychological capital of an entrepreneur goes beyond human capital and social capital, and it also has the characteristics that capital can bring returns to owners. First, the psychological capital of an entrepreneur influences the performance of new ventures by amplifying their entrepreneurial ability and then carrying out efficient entrepreneurial activities or improving the performances of the individual in work. Second, the level of psychological capital of an entrepreneur affects the level of psychological capital of an employee and the performance of new ventures through the improvement of the ability and job performance of an employee. Third, the higher the level of the psychological capital of the entrepreneur is, the stronger their ability to resist the uncertain environment, the higher the survival rate of new ventures is, and the greater the market share they occupy over time, thus, the higher the performance of new ventures is (Deng et al., 2021; Kozlova, 2021).

According to the above contents, hypothesis 3 is obtained and defined as H3:

H3: City economic images have a positive impact on the performance of new ventures.

### Internal Relationship Between the Culture of the City and Its Entrepreneurial Social Capital

Cultural values, as a deep cultural belief system, are the general view of the subject of the subjective and objective world and the relationship between them. It reflects the cognition and relationship of the subject with the state, society, region, or organization, and affects the self-identity, understanding of the meaning of life, and interpersonal relationship of the subject. Cultural values have a guiding role for entrepreneurs. The actions of entrepreneurs with different directions can reflect different cultural values. It can be said that entrepreneurship inherently embodies the cultural values of entrepreneurs. From the perspective of cultural values, it can be found that innovation is not only in the field of scientific and technological discovery and invention but also in the field of values. The innovation of entrepreneurship in cultural values is manifested as the accumulation and growth of cultural capital.

Entrepreneurship is the product of economic and cultural development in modern society. There is no consensus on the definition of the essence of entrepreneurship in academic circles. However, some valuable viewpoints about entrepreneurship are formed after the analysis from different perspectives, and they are the psychological characteristics, the cognitive ability, and the unique quality of entrepreneurs. The connotation of entrepreneurship is defined from the following aspects. First, it refers to innovative spirit, that is, entrepreneurs always search for changes and regard them as an opportunity. The main body of innovation is entrepreneurs and reformers in the economic field, who can boldly break through the traditional business models and practices in the continuous fierce competition, actively mobilize various available production resources, open new markets, and create new organizational forms and business models. In the modernization of today, the sense of hardship, dedication, and hard work of entrepreneurs is the embodiment of professionalism. Second, it is the spirit of cooperation. Enterprises are the product of economic and social development, along with the basic element of the social division of labor and cooperation. Entrepreneurs cannot rely on their efforts to manage all aspects of enterprises and must have a spirit of cooperation so that everyone can work together to form cooperative benefits. With the expansion of the scale and division of enterprises, the entrepreneurial spirit of cooperation is becoming more and more important. The essence of cooperation is that all parties achieve the final synergy effect and a win-win pattern through the replacement, exchange, and coordination of resources and capabilities. Entrepreneurship is always relative because the new concepts will gradually become traditions, and innovation will also change into conventions with the continuous expansion of entrepreneurial cultural values (Xia and Liu, 2021).

Therefore, hypothesis 4 is obtained and defined as H4:

H4: City environmental images have a positive impact on the performance of new ventures.

### Influencing Factors of Entrepreneurial Environment Based on Interpretative Structural Modeling Method

Although domestic and foreign scholars analyze and study the basic characteristics of the entrepreneurial environment, they do not explore the relationship between various entrepreneurial environments, which makes people need to identify the relationship between elements in the process of improving the entrepreneurial environment. Based on this, the previous studies on the entrepreneurial environment are summarized, and ISM is used to study the relationship between the elements of the entrepreneurial environment. It is concluded that the key elements of the entrepreneurial environment provide a theoretical basis for improving the entrepreneurial environment of the country or region by understanding the relationship between the elements at different levels. The idea of ISM is to select the system elements first, then construct the model and establish the adjacency matrix and reachability matrix according to the detailed list of elements. If two factors are influencing each other, the corresponding reduction matrix should be established and hierarchically processed, and the multi-level hierarchical directed graph is drawn according to the matrix obtained by hierarchical structure processing. Finally, the relationship between various factors to be solved can be analyzed according to the directed graph to guide decision-makers to make objective decisions.

The relationship between the influencing factors of the entrepreneurial environment is reflected in five levels. Among them, business and professional infrastructure, entry barriers, physical infrastructure, cultural and social norms, education, and training are the basic elements in the influencing factors of the entrepreneurial environment at the most basic level of ISM. Under the support of this basic element, the second-level basic influencing factors of the entrepreneurial environment, including social and economic conditions, entrepreneurship and business skills, research and development transfer, and intellectual property rights are formed. Under the guarantee of these two basic elements, the second-level basic influencing factors of the entrepreneurial environment, including government policies and regulations, and government projects, are formed.

Thus, the key elements of the entrepreneurial environment include the business environment, education environment, technology environment, policy environment, and city culture. According to its multi-level hierarchical interpretation structure model, it can be found that these six elements have obvious hierarchical characteristics. It is suggested that in the process of improving the entrepreneurial environment, the city cultural environment should be considered at the grassroots level, and the improvement of the city cultural environment will deeply affect the technological environment and economic environment (Iregui et al., 2021).

Based on the above analysis, hypothesis 5 is obtained and defined as H5:

H5: City communication images have a positive impact on the performance of new ventures.

### Design of the Questionnaire on the Influencing Factors of City Image Building

**FIGURE 1:**
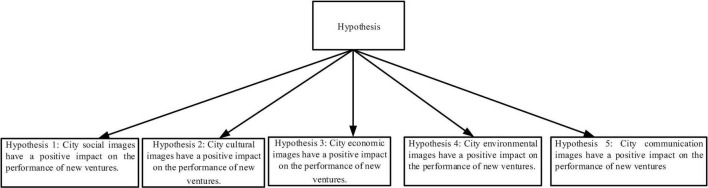
The hypotheses proposed.

Based on the above contents, the study makes an intuitive image representation of the five hypotheses, as shown in Figure 1.

Based on the above five hypotheses, a set of investigation scheme is designed. A city image is not only an objective existence but also a subjective perception. In addition to the objective index of the city, there is also the subjective evaluation of the city image by the audience. Therefore, a more comprehensive evaluation index system of the influence of city image should be built based on the above two survey objectives when the questionnaire is designed, and relevant questions should be set up to achieve the survey task.

The question design of the questionnaire will be divided into five measurement levels according to the influence of each factor on the city image, and the order of the options is “extremely severe – severe – moderate – mild – no influence.” According to the measurement level, the option of “no influence” can help to judge whether the factor is related to the city image. The other four options of “extremely severe; severe; moderate; and mild” can judge the influence of this factor on the city image. Therefore, the question-setting of this questionnaire is helpful to achieve the two objectives of this survey.

In addition, the questions are designed to try to fully interpret the evaluation standard of the influence of the city image from the perspective of the audience. The fields involved in the questionnaire are the influence of the social image, cultural image, economic image, environmental image, and communication image of the city. The elements of social image influence are mainly divided into transportation, medical treatment, housing, education, and social security. Elements of cultural image influence are mainly divided into the library, museum, theater, and concert hall. Elements of economic image influence are mainly divided into tourism, investment, and the number of commercial brands. The elements of environmental image influence are mainly divided into greening, climate, and water source conditions. Lastly, the elements of the influence of communication image are mainly divided into city image advertising, media reports, and city image recognition system. The elements of the above five fields are closely related to the influence of city image and affect the life, study, and work of the audience, as well as the experience and evaluation of city image by the audience. Therefore, this questionnaire can fully show the elements of the influence of city image and make an in-depth investigation on the relationship between these elements and the influence of city image.

Five hundred questionnaires are distributed nationwide, and 456 valid questionnaires are collected through online Q&A, with a recovery rate of 91.2%. According to the data statistics of the collected questionnaires, the proportion of each option is calculated according to the choice of the audience of each influencing factor, and the basic judgment of the audience on the contribution of each factor to the image of the city is analyzed. Then, the scores are set, and the extremely severe is scored 5, severe is 4, moderate is 3, mild is 2, and no influence is 1, respectively. Subsequently, the average score of each factor is calculated according to the percentage of each option, thereby judging the relationship between this factor and the influence of city image. The specific calculation equation is as follows:

(1) Q=5×a+4×b+3×c+2×d+1×e

*Q* is the average score of an impact factor, and *a*, *b*, *c*, *d*, and *e* are “extreme,” “severe,” “moderate,” “mild,” and “no impact,” respectively.

### Validity of the Questionnaire

The effectiveness of the questionnaire directly affects the quality of the survey results. A high-quality questionnaire can more objectively reflect the actual situation. The validity of the questionnaire is usually measured by reliability and validity.

#### Reliability

The reliability of the questionnaire is to identify the consistency and stability of the measurement results. For example, if the test results are consistent in the pre-test and the post-test, the reliability of the questionnaire reliability is higher, and vice versa.

#### Validity

Questionnaire validity is used to reflect whether the survey results can truly reflect the objectives and intentions of the measurement. It is an important index to evaluate the quality of the questionnaire. It is worth noting that validity is a concept of relativity, even if the same measurement result varies with the target. The misunderstanding caused by unclear topics or unclear requirements should be avoided to improve the validity of the measurement in the process of designing the questionnaire.

Reliability and validity are both related and differentiated. If the reliability is high, the validity is not necessarily high, and if the validity is high, the reliability is also high. The pre-questionnaire should be tested before the formal questionnaire is issued to improve the reliability and validity of the questionnaire. The purpose of the pre-test is to find out whether the content structure, logic, language, and other aspects of the questionnaire need to be corrected, and sometimes used to estimate the reliability and validity of the questionnaire. And the statistical software is used to test the recycled pre-test questions. Descriptive statistics are used to explore the position of the subjects, the nature, the size, and the region of the enterprise, which can reflect whether the sample of the questionnaire is representative or not.

#### Item Analysis

Item analysis is to calculate the critical ratio (CR) of each item. The method is that the total score of the item in the pre-test scale is arranged according to the level. The first 27% of the score is the high group, and the last 27% is the low group. The significance test of the difference in the average score of each item between the high and low groups is obtained. If the CR value of the item reaches a significant level, it means that the item can identify the reaction degree of different subjects. This is the first consideration for whether the item should be deleted.

#### Questionnaire Validity Analysis

After item analysis, the structural validity of the scale should be tested, and validity analysis should be carried out. The so-called structural validity refers to the degree to which the scale can measure the concept or characteristics of the theory. Exploratory factor analysis is used to test the goodness of fit between the data and the model, to ensure the structural validity of the questionnaire, and the universality of the questionnaire. That is, the potential structure of the scale should be found out, and the number of topics is reduced to make it a group of having less number and more relevant variables. In the actual investigation and study, the validity construction of the scale sometimes requires factor analysis twice or thrice because in the first-factor analysis of some scales, the content of the topics covered by the factor level is too different, and the explanation at the same level is unreasonable. Therefore, it may be necessary to delete some topics, and the validity of the deleted item needs to be reconstructed. The items with high correlation are screened by factor analysis, and the items with low explanatory power are deleted to ensure the construct validity of the scale. If the rotation of the component matrix in the principal component analysis is quite different from the expected results, it shows that the questionnaire is not reasonable and needs to change the topic and even adjust the structure of the questionnaire.

#### Questionnaire Reliability Analysis

After the factor analysis is completed, the reliability of the scale and the total scale need to be analyzed, that is, the reliability or stability of the scale should be tested. The higher the reliability of a scale is, the more stable the scale is. Generally speaking, the reliability coefficient should be between 0.5 and 0.6.

Table 1 shows the reliability statistics of the questionnaire.

**TABLE 1:** Reliability statistics.

**Cronbach’s alpha**	**Cronbach’s alpha based on standardized items**	**Items**
0.791	0.761	6

Table 1 shows that the reliability of the questionnaire is tested by the internal consistency of Cronbach’s Alpha reliability coefficient, which ensures the consistency of the scoring direction of each item option. The measured value of α is 0.797, indicating that the questionnaire has good stability and internal consistency.

## Investigation and Analysis on the Influencing Factors of City Image

### Influence of City Images on Entrepreneurs

**FIGURE 2:**
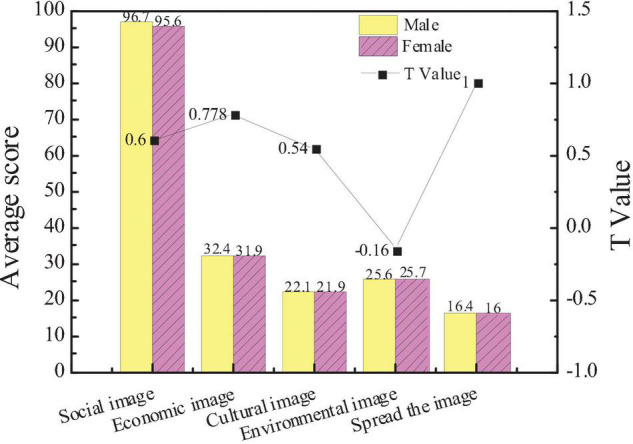
Comparison of the attention to urban images between male and female entrepreneurs.

According to the survey results, male and female entrepreneurs are different in the attention to city images, as shown in Figure 2.

In terms of the city social image, the average score of females is 95.6, and that of males is 96.7. In terms of the city economic image, the average score of females is 32.4, and that of males is 31.9. In terms of economic images, the environmental image, and the communication image, the average score of females and males is the same. The results show that there is no significant difference in the attention of entrepreneurs of different genders to city images (*p* > 0.05), and most entrepreneurs pay more attention to the social image than the communication image.

**FIGURE 3:**
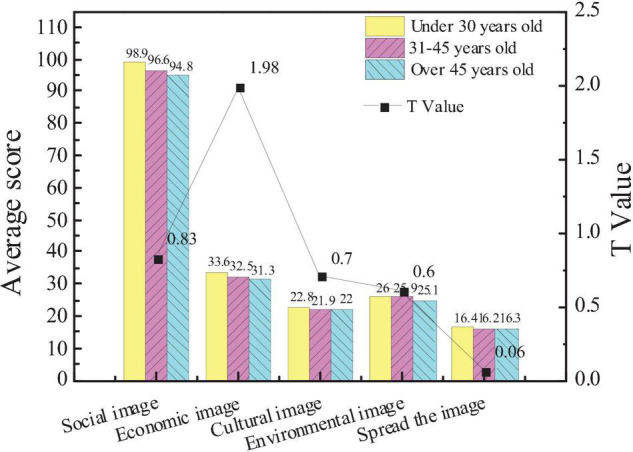
Comparison of the attention of the entrepreneurs of different ages to the city image.

Figure 3 shows the comparison of the attention of the entrepreneurs of different ages to the five aspects of the city image.

The average score of the subjects under the age of 30 in the city social image is 98.9, that of the subjects aged 31–45 is 96.6, and that of the subjects over 45 is 94.8. On the impact of the city economic image, the average score of the subjects under 30 years old is 33.6, that of the subjects aged 31–45 is 32.5, and that of the subjects over 45 is 31.3. The results show that there is no significant difference in the attention of entrepreneurs of different ages to different dimensions of the city image (*p* > 0.05), indicating that age has little effect on entrepreneurial social capital.

**FIGURE 4:**
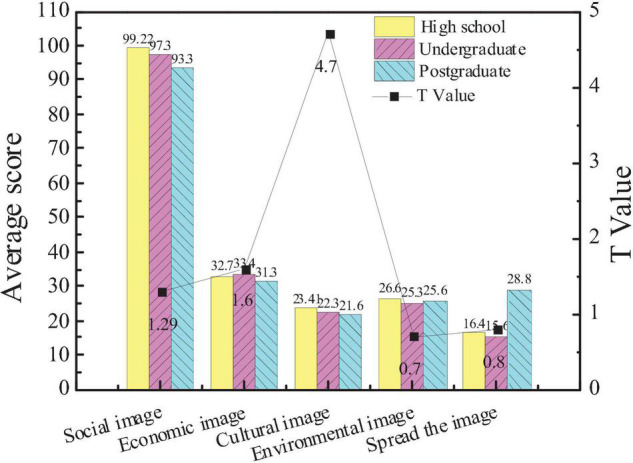
Comparison of the attention of the entrepreneurs with different educational backgrounds to the city image.

Figure 4 shows the attention of the entrepreneurs with different educational backgrounds to the five aspects of the city image.

The average score of the subjects with high school education in city social image is 99.22, that of the subjects with undergraduate education is 97.3, and that of the subjects with graduate education is 93.3. The average score of the subjects with high school education in the city cultural image is 23.4, that of the subjects with undergraduate education is 22.3, and that of the subjects with graduate education is 21.6. The data show that there are significant differences in the attention of entrepreneurs with different educational backgrounds to city images (*p* < 0.05), but there is no significant difference in the other four aspects. Moreover, the higher the educational backgrounds are, the more the attention to the cultural image of the city is.

### A Survey on the Influencing Factors of City Social Image

**FIGURE 5:**
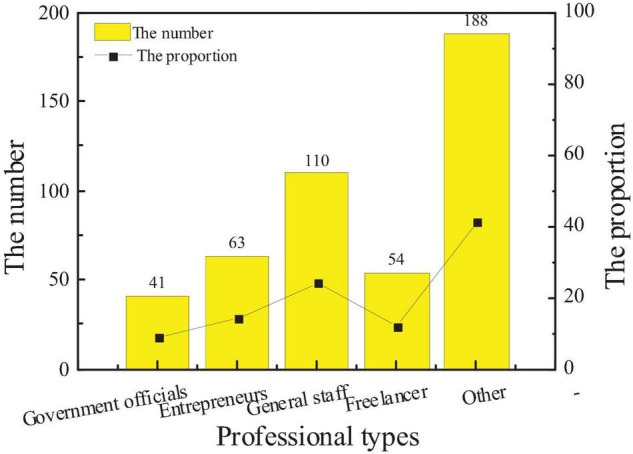
Distribution of professional types of interviewees.

After the results of this questionnaire are sorted out, the interviewees are roughly divided according to the five basic types of government officials, entrepreneurs, general staff, freelancers, and others. The specific distribution of the number is shown in Figure 5.

The data show that the main occupations of the surveyed population are ordinary workers, followed by entrepreneurs and government officials, and the least is freelancers. There are 41 government officials, 63 entrepreneurs, 110 ordinary employees, and 54 freelancers. The study finds that 62% of entrepreneurs are new entrepreneurs, and their sensitivity to the city brand image is slightly higher than that of other types of occupations.

**FIGURE 6:**
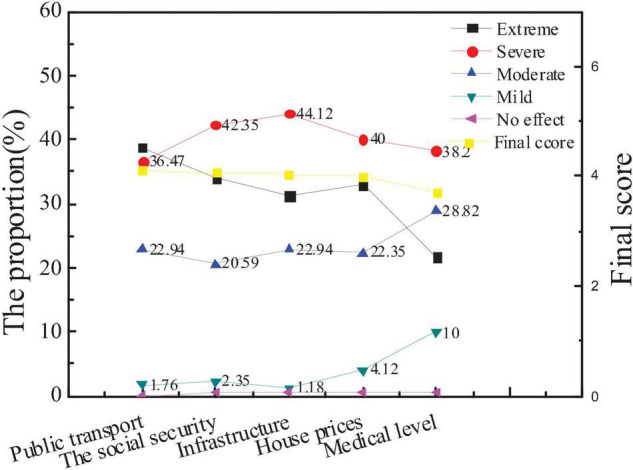
Attention and scores of the audiences on the influencing factors of city social image.

**FIGURE 7:**
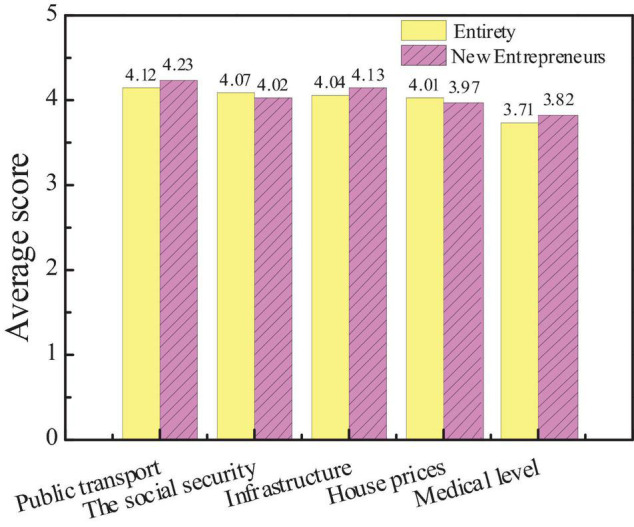
Comparison between the average scores of new entrepreneurs and the overall audience.

Specifically, the survey results of the influencing factors of city social image and the average score of each influencing factor of new entrepreneurs for city social influence are compared with the overall data. The results are shown in Figures 6, 7.

The figure shows that city public transport has the greatest impact on city social image, with an average score of 4.12. The medical level has the least impact on the social image, with an average score of 3.71. The social environment affects the judgment of the audience of the influence of city image. Good security conditions, good social welfare and security system, and convenient transportation facilities not only affect the quality of life of residents but also act as an important evaluation index. After the comparison is conducted, there is little difference in the concern of new entrepreneurs for various influencing factors from the overall situation, but the average score of public transportation and infrastructure construction is significantly higher than that of the overall situation, indicating that new entrepreneurs are more concerned about the transportation and infrastructure construction than ordinary people. And Hypothesis 1 is supported.

### Survey Results on Influencing Factors of City Cultural Image

**FIGURE 8:**
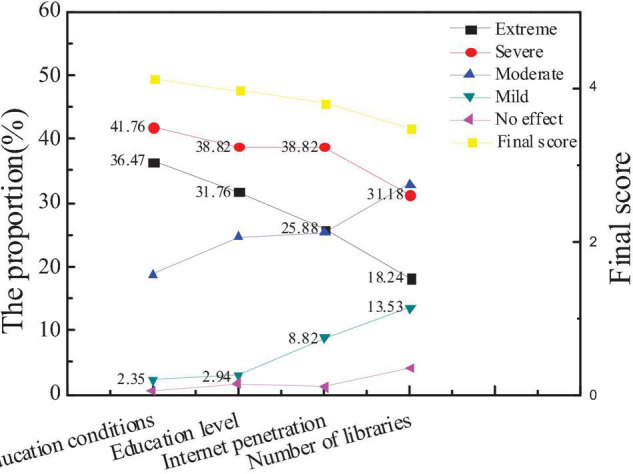
Attention and scores of the audiences on influencing factors of city environmental image.

**FIGURE 9:**
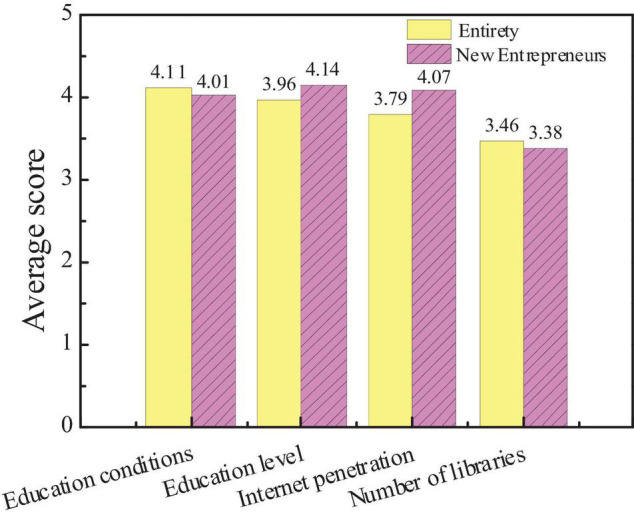
Comparison of the average scores between new entrepreneurs and the overall audience.

The survey on the influencing factors of city cultural image is mainly divided into city education conditions, the overall education level of residents, the popularity of the Internet, and the number of libraries. Based on the statistics of the questionnaire, the public and new entrepreneurs pay more attention to the influencing factors of city cultural image, as shown in Figures 8, 9.

Figures 8, 9 show that the average score of the city education level is slightly higher than the education level of residents, the network popularization, and the number of libraries is more important to the cultural image of the city. The average score, education level, and Internet popularity rate of new entrepreneurs are higher than the overall average score, indicating that the public believes that the educational conditions of the city are the most important to constructing the cultural image of the city. However, new entrepreneurs believe that the education level of urban residents and the popularity of the Internet can better reflect the cultural image of a city. Hypothesis 2 is verified.

### Survey Results on Influencing Factors of City Economic Image

**FIGURE 10:**
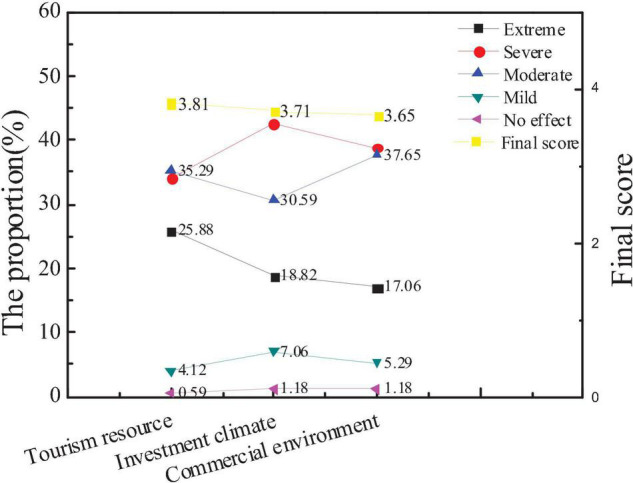
Attention and scores of the audiences on the influencing factors of city economic image.

**FIGURE 11:**
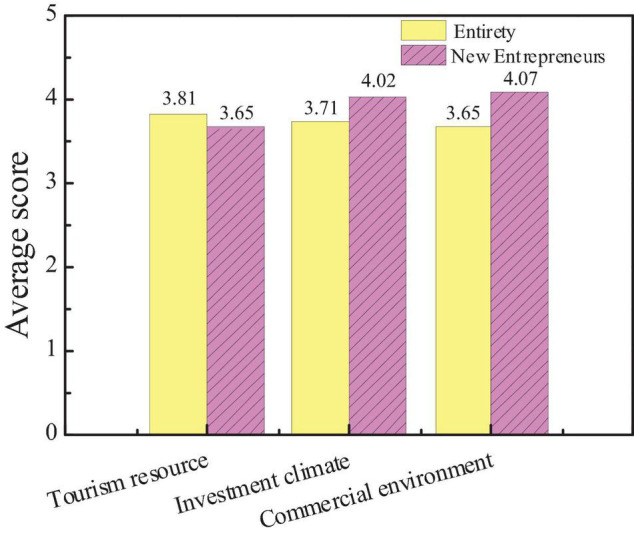
Comparison between the average scores of new entrepreneurs and the overall audience.

The survey on the influence of city economic image is mainly divided into the survey of tourism, investment, and the number of commercial brands. Based on the data statistics of the questionnaire, the attention of the public to different factors and the average score of new entrepreneurs are compared, as shown in Figures 10, 11.

The figure shows that the public pays more attention to city tourism resources than investment climate and commercial environment, and the average score of tourism resources is also higher than the other two. This shows that the public generally believes that the economic image of the city is most closely related to city tourism resources, but the average score of new entrepreneurs on the three factors is opposite. The average score of investment climate and commercial environment is higher, indicating that the investment climate and commercial environment are more important for new entrepreneurs. And hypothesis 3 is not true.

### Survey Results on Influencing Factors of City Environmental Image

**FIGURE 12:**
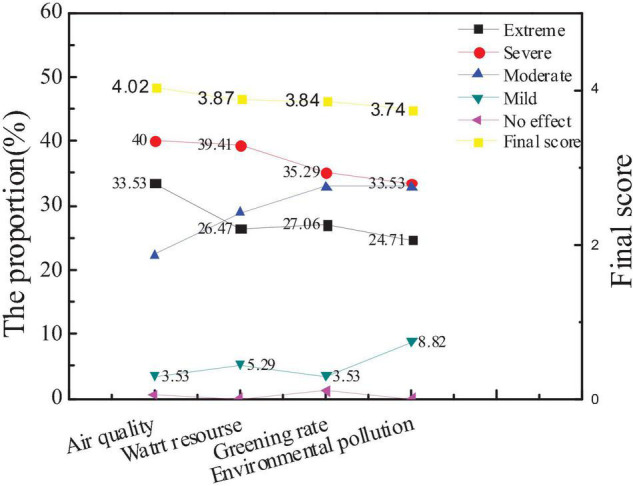
Attention and scores of the audiences on influencing factors of city environmental image.

**FIGURE 13:**
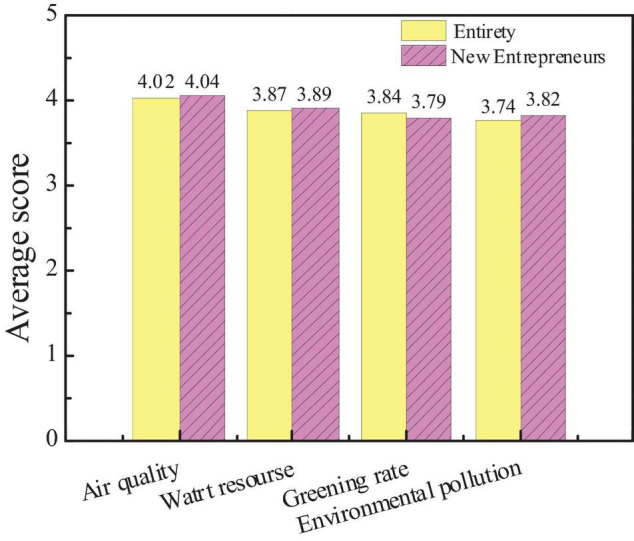
Comparison between the average scores of new entrepreneurs and the overall audience.

The survey on the influencing factors of city environmental image is mainly carried out from air quality, water resources, greening rate, and environmental pollution. Through the data statistics of the questionnaire, the attention of the public to the factors and the average score of new entrepreneurs are compared, as shown in Figures 12, 13.

The figures show that the audience attaches great importance to air quality and water resources, which is consistent with the views of new entrepreneurs. However, the difference is that new entrepreneurs give more attention to the factor of environmental pollution, indicating that the image of the city environment depends largely on air and water quality. Besides, environmental pollution is also a manifestation of the overall environment and governance capacity of a city. And hypothesis 4 is supported.

### Survey Results of Influencing Factors of City Communication Image

**FIGURE 14:**
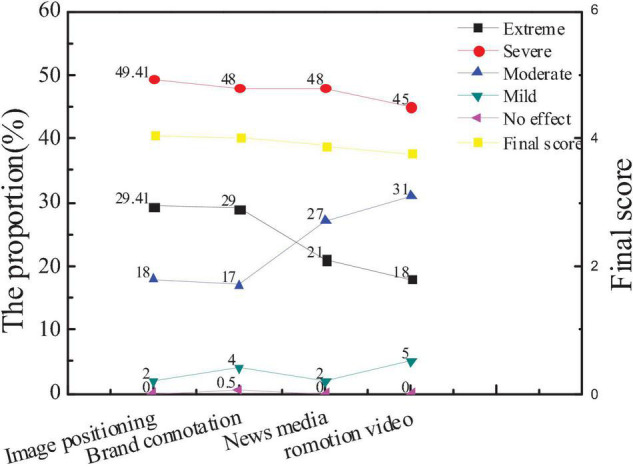
Attention of the audiences to influencing factors of city communication image.

**FIGURE 15:**
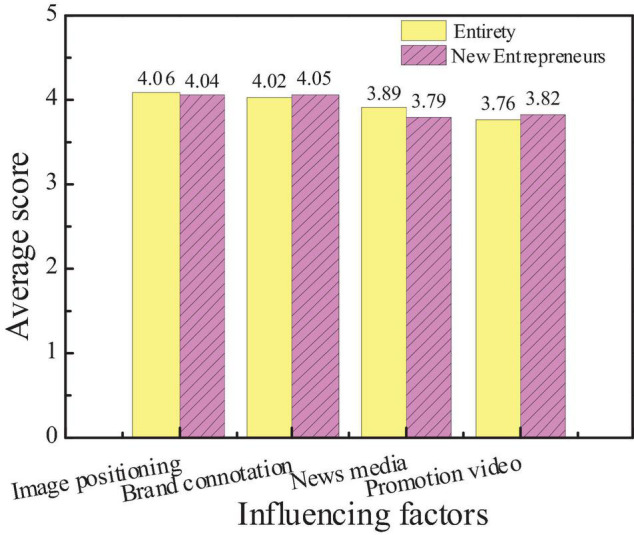
Comparison between the average scores of new entrepreneurs and the overall audience.

The survey on the factors that constitute the image power of city communication is mainly concerned with image positioning, brand connotation, news media, and promotion videos. The survey on the image recognition system, image advertising, and media reports is conducted. Based on the statistics of the collected questionnaires, the attention of the public to different factors and the average score of new entrepreneurs are shown in Figures 14, 15.

The figures show that the attention of the public to the city image positioning and brand connotation is higher than that of the new media and promotion videos, which has no big difference in the comparison with the new entrepreneurs. The average score of the new entrepreneurs in the brand connotation and promotion videos is slightly higher than the overall score, indicating that the new entrepreneurs are more aware of the brand effect and promotion of the city, which is very important to popularize the city. And hypothesis 5 is supported.

## Data Availability Statement

The raw data supporting the conclusions of this article will be made available by the authors, without undue reservation.

## Ethics Statement

The studies involving human participants were reviewed and approved by Chongqing Institute of Engineering Ethics Committee. The patients/participants provided their written informed consent to participate in this study. Written informed consent was obtained from the individual(s) for the publication of any potentially identifiable images or data included in this article.

## Author Contributions

All authors listed have made a substantial, direct and intellectual contribution to the work, and approved it for publication.

## Conflict of Interest

The authors declare that the research was conducted in the absence of any commercial or financial relationships that could be construed as a potential conflict of interest.

## Publisher’s Note

All claims expressed in this article are solely those of the authors and do not necessarily represent those of their affiliated organizations, or those of the publisher, the editors and the reviewers. Any product that may be evaluated in this article, or claim that may be made by its manufacturer, is not guaranteed or endorsed by the publisher.
